# Inborn Errors of Immunity in the Premature Infant: Challenges in Recognition and Diagnosis

**DOI:** 10.3389/fimmu.2021.758373

**Published:** 2021-12-24

**Authors:** Scott M. Gordon, Amy E. O’Connell

**Affiliations:** ^1^ Division of Neonatology, Children’s Hospital of Philadelphia, and Department of Pediatrics, Perelman School of Medicine, University of Pennsylvania, Philadelphia, PA, United States; ^2^ Division of Newborn Medicine, Boston Children’s Hospital, and Department of Pediatrics, Harvard Medical School, Boston, MA, United States

**Keywords:** inborn errors of immunity, primary immunodeficiency, prematurity, infection, necrotizing entercolitis, herpes simplex virus, sepsis, autoinflamatory diseases

## Abstract

Due to heightened awareness and advanced genetic tools, inborn errors of immunity (IEI) are increasingly recognized in children. However, diagnosing of IEI in premature infants is challenging and, subsequently, reports of IEI in premature infants remain rare. This review focuses on how common disorders of prematurity, such as sepsis, necrotizing enterocolitis, and bronchopulmonary dysplasia, can clinically overlap with presenting signs of IEI. We present four recent cases from a single neonatal intensive care unit that highlight diagnostic dilemmas facing neonatologists and clinical immunologists when considering IEI in preterm infants. Finally, we present a conceptual framework for when to consider IEI in premature infants and a guide to initial workup of premature infants suspected of having IEI.

## Introduction

Regulation and dysregulation of immunity in growing premature infants remain poorly understood. Premature infants have immature immune systems, including diminished neutrophil numbers and function, T cell lymphopenia, NK cell dysfunction, and relative hypogammaglobulinemia (1–4). Many of the organ-specific disorders seen in prematurity, such as necrotizing enterocolitis (NEC) and bronchopulmonary dysplasia (BPD), are associated with immune dysregulation including altered cytokine production and skewing of T cell populations (5, 6). Clinical manifestations of such conditions are non-specific and may be indistinguishable from organ-specific manifestations of inborn errors of immunity (IEI), encompassing an array of genetic immunodeficiencies and hyper-inflammatory disorders (7).

Consistent with dynamic, rapid changes in composition and function of the immune system in newborns (8, 9), multiple reports have outlined potential pitfalls of interpreting standard state newborn screening for severe combined immunodeficiency (SCID) in those born preterm (10, 11). No standards of care have been established for the clinical immunologist or neonatologist to guide comprehensive workup for IEI in the extremely premature population. Enhanced awareness of the potential to encounter IEI in the neonatal intensive care unit (NICU), as well as formal collaborations between neonatologists and clinical experts in identifying and treating neonatal IEI, will benefit this often-overlooked population. The goal of this review is to educate the clinical immunologist on common clinical features of prematurity which may suggest IEI in certain circumstances. For a review on features of IEI that can present in the neonatal and premature period, please see our previous review (12). Prompt recognition of these disorders in critically ill, preterm neonates will not only enhance our basic understanding of neonatal immunity but will also accelerate development of novel management strategies and therapies for affected infants. IEIs individually are rare, but collectively are not. Reports of IEI in premature infants are uncommon, suggesting that IEI may be underdiagnosed in premature infants and highlighting the need for better detection.

## Clinical Vignettes

The following cases illustrate challenges in diagnosis of immunodeficiency and genetic inflammatory disorders in premature infants. They are not meant to suggest that each neonate presented has an IEI, but to illustrate the clinical complexity that can occur when trying to determine whether a premature infant has abnormal immunity.

## Case 1 – Non-SCID T Cell Dysfunction in a Premature Infant

An extremely premature male infant born at 24 weeks gestation developed severe NEC with poor wound healing and required a wound vacuum for several months in order for the abdomen to fully heal. The patient also had recurrent *Candida parapsilosis* infection with bloodstream and urinary tract infection, as well as severe thrush. Initial immune testing at 30 weeks gestation showed severe T cell lymphopenia but was attributed to the patient’s immaturity and clinical status at that time. The initial SCID screen was reported as normal at 7 days of age by the referring hospital but was persistently abnormal (<252 copies/uL) at our hospital from 1 month on. Repeat immunologic testing at 41 weeks postmenstrual age (neonatal nomenclature for the corrected gestational age accounting for age at birth plus weeks since birth) demonstrated T cell lymphopenia and abnormal T cell proliferation, but the patient became abruptly ill the following week and died from *Klebsiella* sepsis. Whole exome testing and protein analysis revealed heterozygous null mutations in *OSBPL5* in trans, a gene not before implicated in human disease that has been shown to be important for CD4 T cell proliferation in humans (13). Phenotypic and mechanistic evaluation is ongoing in the laboratory. In this situation, the patient’s prematurity and severity of illness clouded the ability to evaluate for IEI. Earlier use of rapid exome testing may have been clinically beneficial.

## Case 2 – Complement Variants in a Premature Infant With Recurrent Infections

An infant born at 25 weeks’ gestation developed expected complications of prematurity, including chronic lung disease and surgical NEC. At 4 months of age, she abruptly decompensated with cardiorespiratory failure and required extracorporeal membrane oxygenation (ECMO). She was found to have an abdominal abscess and *Pseudomonas* sepsis. Standard immune testing showed mild T cell lymphopenia but was otherwise unremarkable. CH50 and AH50 levels were normal. Genetic testing, however, showed a mutation in *MBL2* (mannose binding lectin) that was predicted to be pathogenic. *MBL2* defects have been shown to increase susceptibility to infection in infants (14), and this genetic disorder may particularly enhance infectious susceptibility in the time period before the baby begins to produce its own IgG in significant amounts. Premature infants with MBL2 polymorphisms have an increased risk for adverse neurological outcomes (odds ratio of 8.67) compared to infants without variants (15). The patient recovered from the initial infection, but she later developed recurrent *Pseudomonas* peritonitis several months later. This case illustrates how apparent complications of BPD or NEC can be difficult to discern from abnormal infections that signal genetic variants in complement and humoral immunity.

## Case 3 – Severe Infection From Prematurity Without an IEI

An infant born at 32 weeks gestation developed early-onset *Escherichia coli* meningitis and sepsis on day of life 1. Despite rapid recognition and treatment with appropriate antibiotics, the patient developed multiple intracranial empyemas that were not amenable to direct drainage. The patient completed seven weeks of antibiotics but exhibited severe loss of brain parenchymal tissue with development of pervasive seizure disorder. The patient again had recurrence of *E. coli* sepsis and meningitis at 2 and a half months of age, despite negative cultures on multiple occasions in the interval between the first and second infections. Immune testing was normal including a T/B/NK cells and T cell memory panel except for decreased absolute NK cell number (94 cells/uL, ref range 160-1100), and abnormal T cell proliferation to anti-CD3 stimulation (3723 CPM, ref >62,927; background was normal). Trio whole exome sequencing was sent and revealed no convincing candidate genes. *E. coli* is a well characterized cause of neonatal meningitis, and certain virulence factors are associated with increased risk for neonatal meningitis, such as the presence of the K1 capsule. Gram negative meningitis in premature infants is often devastating neurologically and can cause significant brain volume loss. Severe outcomes have been tied to virulence factors such as the *E. Coli* K antigen.

## Case 4 – Autoinflammatory Disease in a Premature Infant

A 33-week premature infant was born in the setting of preterm labor. The infant had mild respiratory distress but also had multilineage cytopenias, significant liver dysfunction with coagulopathy, conjugated hyperbilirubinemia, transaminitis, and a ferritin level that peaked over 30,000. Soluble IL-2R level was normal. Her liver MRI was inconsistent with neonatal hemochromatosis (also referred to as gestational autoimmune liver disease/GALD”). Mean fluorescence staining for perforin in NK cells was low. The team was unable to obtain a suitable bone marrow biopsy specimen. Given continued disease progression and suspicion of primary hemophagocytic lymphohistiocytosis (HLH) based on low perforin expression, she was treated with IVIG (with little impact) and then transitioned to steroids. Her ferritin began to decrease with initiation of steroids. Unable to wean steroids without relapse, she eventually was transitioned to etoposide and cyclosporine. She was discharged from the NICU around 2 months of age. Specific gene-based testing for common genetic causes of HLH and whole exome sequencing was non-diagnostic. She went on to have a bone marrow transplant within the first year of life. This case illustrates the clinical overlap between hemophagocytic autoinflammatory disorders and neonatal disorders caused by maternal immune activation.

## Case Series Conclusions

All of these patients were cared for in a single tertiary NICU within the past three years. Several of these infants did have IEI or clinically important polymorphisms in immune genes. If our unit is similar to other regional referral NICUs, it suggests that IEI may be underappreciated in the premature population. The immune interface is critically important during gestation, and perturbation of the maternal-fetal immune milieu by IEI might actually lead these individuals to be overrepresented in the NICU compared to term infants – it is simply not known.

These cases reinforce how difficult it can be to discern prematurity-related immune immaturity from genetic causes of primary immunodeficiency. Because something is challenging, however, does not mean it is impossible. In the age of cost-effective and rapid genetic testing, especially whole exome sequencing, this presumption of diagnostic helplessness must be reconsidered. Early detection of IEI saves lives, and in some cases, routine enhanced precautions and antibiotics may allow for a bridge to more definitive treatment once the patients are mature and stable enough for bone marrow transplantation or gene therapy. In addition, many of these families will go on to attempt to have additional children, in which case identifying genetic risk can help with family counseling and preimplantation diagnostic efforts.

## Clinical Features of Prematurity Which May Suggest IEI

### Infections

A hallmark of IEI is an abnormal host response to infection, leading to life-threatening disease in infancy and beyond (7). Premature infants have immature immune systems and can be considered relatively immunocompromised compared to older children and adults in terms of both innate and adaptive immune responses (16). Immature immunity is one reason premature infants are susceptible to a variety of severe bacterial, fungal, and viral infections. Given the overlap between secondary immunodeficiency of prematurity and IEI, clinicians in the NICU must maintain a high index of suspicion for IEI in the setting of recurrent or unremitting infections and inflammation (**Table 1**).

**Table 1:** Features of prematurity that may suggest IEI.

Clinical Scenario	Reason to Consider IEI	Suggested Evaluation
Severe HSV	Mutations in TLR3, the complement pathway, and *STAT1* have been seen	Whole exome sequencing (WES). (*Specific tests for these pathways are not currently available*).
RSV requiring ECMO or recurrent severe RSV	T cell immunodeficiencies have been associated with severe disease.	T/B/NK cell panel, consider SCID genetic panel if abnormal.
Recurrent severe bacterial infections *or* Unusual bacterial pathogens *or* Unusual bacterial infection sites	Can be a hallmark of a wide variety of IEIs	Consider T/B/NK cell panel, mitogen testing, DHR testing for CGD, immunoglobulins, additional functional and genetic testing depending on scenario
Hepatomegaly with cytopenias	Can be seen with autoinflammatory disorders	Inflammasomopathy panel*, HLH gene panel
Necrotizing enterocolitis – recurrent or with prolonged wound healing	NEC has immunologic component, severe cases might suggest IEI	Consider T/B/NK cell panel, mitogen testing, DHR testing for CGD, immunoglobulins; consider WES
Persistent diarrhea or colitis	VEO-IBD and a variety of other IEIs can present in neonatal period.	VEO-IBD genetic panel, genetic screening for congenital diarrheas. Also consider genetic testing for autoinflammatory diseases, IPEX, XIAP.
Bronchopulmonary dysplasia	Not a clear link with IEI, but BPD may cause thymic atrophy and secondary T cell lymphopenia	Consider immune evaluation for recurrent sinopulmonary infections (Igs, B cells, CGD, complement)
Low T cell receptor excision circles (TRECs)	Hallmark of severe combined immunodeficiency	Repeat up to twice in premature infants, if still abnormal do T/B/NK cell panel. If flow cytometry is abnormal, equivocal, or unable to be done move to genetic testing or WES.

*For example, Invitae offers an Autoinflammatory and Autoimmunity Syndromes Panel that includes 115 different genes associated with inflammasomopathies and autoinflammatory disorders. The authors do not endorse any particular lab or vendor.

### Viral Disease

Herpesviral infections have especially high morbidity and mortality in the newborn period due to the increased incidence of disseminated HSV and CNS disease (17). Anti-herpesviral immunity is complex, with key roles played by barrier immunity, complement, pattern recognition receptors (e.g. Toll-like receptor [TLR] 3, RIG-I-like receptors), myeloid cells (e.g. plasmacytoid dendritic cells), lymphocytes (T and B cells, natural killer [NK] cells), and antiviral cytokines (e.g. interferons) (18). Indeed, severe herpesviral infections are seen in IEI, especially with mutations affecting NK cells and components of the TLR3 signaling cascade (19, 20). Recently, immune- and CNS-related genetic variants have been reported in association with neonatal HSV, including skin-eye-mouth, disseminated, and CNS disease (21). Similar to mutations observed in older patients, mutations in TLR3, as well as the downstream mediators TRAF3 and IRF3, were found by whole exome sequencing in this cohort of neonates with severe HSV disease. Mutations were also found in genes encoding complement (*C6* and *C7*), the double-stranded RNA sensor *MSR1*, the cytolytic mediator *PRF1*, *RAG2* (also associated with SCID), the interferon-stimulated transcription factor *STAT1*, and the antiviral proteins *GRB2* and *DBR1*. Neonatal HSV is rare in the current era of perinatal adaptations to diminish HSV transmission, which include routinely prescribing prophylaxis to mothers with a history of genital herpesvirus carriage and recommending cesarean section for mothers with active lesions at the time of delivery (17, 22). Thus, evaluation for IEI should be considered in the infant who presents with severe neonatal HSV (**Table 1**), especially in the absence of overt risk factors such as a maternal history of active HSV. As susceptibility to HSV may be driven by mutations in pathways for which reliable clinical immunologic tests do not exist (particularly for premature infants), we suggest moving directly to comprehensive genetic testing in these cases. Even a rapid genetic diagnosis may not alter the course of HSV disease. However, we believe it is essential to make an accurate diagnosis to guide long-term interventions, such as need for future bone marrow transplantation, for the infant who survives this disease. We also believe in the value of providing closure to families in the unfortunate case of death from neonatal HSV, as well as critical information for future family planning.

Infants who contract respiratory syncytial virus (RSV) and require advanced respiratory support are commonly encountered in Level IV NICUs that serve as referral centers and admit patients from home. Former preterm infants, especially those diagnosed with bronchopulmonary dysplasia (BPD), are particularly susceptible to severe disease requiring hospitalization (23). Similarly, IEIs that impact cellular immunity, such as a variety of SCID variants, CD40L deficiency, Omenn Syndrome, and MHC Class II deficiency are associated with severe RSV-mediated disease (24, 25). Lower respiratory infection with RSV causes millions of hospitalizations per year. Thus, it would be impractical to screen for IEI in every infant hospitalized with RSV. However, abnormalities of cellular immunity are well-known to increase susceptibility to severe RSV disease in infants (26, 27). While humoral immunity is clearly important in the response to RSV, presentation of humoral immunodeficiency would be unusual prior to 4-6 months of age, when transplacentally acquired maternal IgG wanes. We suggest comprehensive assessment of cellular immune composition and function, with WES to also be considered, in those infants suffering recurrent or severe disease (having more than one RSV hospitalization or needing ECMO, for instance (**Table 1**).

Several other viruses also have a predilection for both premature infants and children with IEI and can present with ocular disease (28). Enteroviral retinopathy and CMV retinitis have been observed in full-term infants with SCID (29, 30). Preterm infants are routinely surveyed for retinopathy of prematurity (ROP) while in the NICU. Findings incongruent with classic ROP should be evaluated and evidence of retinitis in the setting of other signs such as abnormal newborn screening or recurrent infections would raise suspicion for IEI.

### Bacterial and Fungal Disease

Several forms of IEI present in the neonatal period with unusual or recurrent bacterial and/or fungal infections (31). While these infections are alarming in previously healthy, full-term neonates, they can be common in the NICU. Critically ill neonates exhibit impaired barrier function due to immaturity and the need for indwelling devices, such as endotracheal tubes and central venous catheters. Invasive bacterial and fungal infections in the NICU are typically attributed to incomplete development of the neonatal immune system and increased exposure risk. IEI should be considered for recurrent culture-proven bacterial sepsis (**Table 1**) (32).

Given that both prematurity and IEI can independently lead to recurrent bacterial infections, it can be challenging to know when infection in a premature infant should prompt immunologic evaluation. We recommend that infants with recurrent, culture-positive bloodstream infections not thought to be due to an indwelling central line be evaluated for IEI with immunologic screening. Likewise, premature infants who develop severe infections in unusual locations or with unusual pathogens should be screened for IEI. While determining what is unusual may at times be nuanced, there are several helpful reviews that cover the types of unusual infection characteristics that would prompt an evaluation for IEI (33–36). Premature infants with recurrent tracheitis, bacteremia in the setting of NEC, or less than three bloodstream infections associated with indwelling central lines and responsive to antibiotics should not raise additional suspicion for IEI. Research-based evidence is lacking in terms of distinguishing abnormal from abnormal infections in premature infants, but in our clinical experience certain infection features align more with prematurity and others are more concerning for IEI. We have outlined our suggestions in **Table 2**.

**Table 2:** Clinical infectious history and risk of IEI in premature infants.

Infection History	Clinical Guidance
Recurrent tracheitis or ventilator-associated pneumonia	Very common in premature infants (37). Would only consider IEI if patient has recurrent respiratory infections where they become unusually severely ill, require ECMO, etc. *Staph aureus*, particularly MRSA, can cause severe respiratory disease in premature infants including necrotizing pneumonia, so an isolated severe respiratory infection with *Staph aureus* (with or without bacteremia) would not raise suspicion of IEI (38).
Recurrent sepsis	20% of extremely premature infants (<29weeks) will have sepsis during their NICU admission (39). It is unusual, though, to have multiple bouts of sepsis with different organisms. This should prompt consideration for IEI evaluation.*
Fungal infection	Candidal infections are common in premature infants (40). However in our experience a persistent fungal infection not responding well to appropriate antifungals should prompt consideration of defects of cellular immunity. Other types of fungal infections (*Aspergillus*, Zygomycetes, *Malessezia*) are extremely uncommon despite being ubiquitous in the environment. Unusual fungal infections should prompt a broader IEI evaluation.
Unusual bacteria	Gram positive infections are the most common causes of late onset sepsis in the NICU. Atypical gram positive organisms like *Nocardia* are exceedingly rare in premature infants. Among gram negatives, *E. Coli*, *Klebsiella* sp., *Serratia* sp. and Enterobacter sp. are the most common causes of sepsis. Infections outside of these common groups should prompt further consideration*.
Unusual infection sites	Bloodstream infections, pneumonia, urinary tract infections, meningitis, osteomyelitis, and intraabdominal abscesses (in the setting of necrotizing enterocolitis) are all relatively common in premature infants. Abscesses in other locations and disseminated bacterial skin infections are unusual and evaluation for IEI should be considered*.
Osteomyelitis or septic arthritis	Typical of premature infants, particularly if *Staph* species or another gram positive organism like Group B strep (41). Consider IEI* with gram negative organism as this is uncommon (especially *Salmonella* which is associated with CGD).
*E. Coli* meningitis	*E. Coli* typically causes very severe CNS disease in premature infants and neonates, often with abscess formation or tissue destruction. It also has a propensity to recur after treatment (42). Studies have shown this is more related to bacterial virulence factors and an immature host than to abnormal immunity (43).

*Consider evaluation for IEI after reviewing the case context and whether there are clear contributing factors to infection or other concerns for genetic disease (nosocomial outbreak, sick parental contact, other concerning clinical features of the infant, etc).

### Neonatal Autoinflammatory Disorders

Autoinflammatory disorders are marked by non-specific activation of inflammatory immune pathways (44, 45). Some autoinflammatory conditions presenting in the neonatal period are caused by activating mutations in inflammasomes, which assemble to promote release of active proinflammatory interleukin-1-beta (IL-1β) and IL-1 (46, 47). One such inflammasomopathy, neonatal-onset multisystem inflammatory disease (NOMID), results from mutation of the NLRP3 inflammasome and presents with debilitating chronic inflammation of the skin, joints, and central nervous system and can present in neonates (48). Mutations of the NLRC4 inflammasome can lead to acute hyperinflammation and macrophage activation syndrome during infancy (49–51). The resultant severe multisystem inflammation can be fatal and can be difficult to distinguish from neonatal sepsis (49, 52).

Several autoinflammatory disorders can lead to macrophage activation syndrome (MAS), which shares a clinical spectrum with hemophagocytic lymphohistiocytosis (HLH). HLH is an autoinflammatory disorder which itself can be caused by specific genetic mutations which are not inflammasome components. Primary HLH is caused by IEIs, while familial HLH is due to non-IEI genetic disorders that affect granule-mediated cytotoxicity by lymphocytes (53). These disorders, MAS and HLH, are marked by cytopenias, fevers, hepatosplenomegaly and increases in acute phase reactants, and they can present in the newborn period and have been documented in premature infants as young as 23 weeks gestation (54, 55). Neonatal hemochromatosis (NH, also called gestational alloimmune liver disease) is due to placentally transferred maternal antibodies and can also occur in premature infants. NH features liver disease, cytopenias, and altered fibrinogen levels and can be difficult to clinically distinguish from HLH (56, 57). In these cases MRI to look for iron deposition (seen in NH) and genetic testing can be helpful.

Hyperinflammatory disease exacerbations, including HLH and inflammasomopathies, can be triggered by infection, making diagnosis challenging in these patients when there is a concurrent infection. Inflammasomopathies should be considered in the differential diagnosis of exceptionally sick preterm infants with cytopenias and evidence of persistently elevated acute phase reactants that do not respond to antibiotic therapy, both in the setting of recurrent “culture negative” sepsis and when an infectious etiology is identified.

### Disorders of the Digestive Tract

The digestive tract is frequently affected in the setting of IEI through a variety of mechanisms (58). Clinical presentations of feeding intolerance, bloody stools, diarrhea, and failure to thrive can manifest in the premature neonate and overlap with signs that may be seen in IEI. In the premature infants these findings are often attributed to impaired structure and function of the underdeveloped digestive tract (59).

Necrotizing enterocolitis (NEC) is a disorder of the neonatal intestine that affects predominantly preterm neonates. NEC is not well understood, occurs without warning, and can be rapidly progressive with high morbidity and mortality (60, 61). NEC is diagnosed with a combination of nonspecific clinical, radiographic, and laboratory findings so that, especially in mild cases, it can be indistinguishable from other types of enterocolitis and sepsis. As investigators have probed genetic evaluation of NEC, NEC has been associated with several susceptibility genes and some of these are in the inflammatory pathway such as Toll-like receptor 4 (TLR4) (62, 63). Dysregulated TLR signaling has also been implicated in premature infants with severe NEC and mutations in *SIGIRR*, a negative regulator of TLRs (64, 65). NEC has also occurred in a patient with Omenn Syndrome, characterized by numerous autoinflammatory manifestations (66). It is impractical to evaluate for IEI in every infant who develops NEC of any severity. However, these data support the notion that NEC can present due to primary immunodysregulation. Special consideration should be given to ELBW infants with recurrent or extreme cases of NEC, as well as otherwise-healthy term infants who develop NEC, especially if the patient also has an unusual infectious history (**Table 1**).

Other types of colitis can present in premature infants, as well, and are often associated with monogenic disorders and IEIs in particular. A growing list of genetic differences resulting in IEI have been linked to development of very early-onset inflammatory bowel disease (VEO-IBD) (67, 68). VEO-IBD can present as early as the first week of life in a similar manner to NEC, however it is persistent in nature while NEC is transient. It can be challenging to manage, with some cases altogether refractory to immunomodulation and supportive therapy. The importance of establishing a definitive diagnosis in such infants cannot be overstated, as hematopoietic stem cell transplant can be curative for several different types of VEO-IBD (69). VEO-IBD falls within the larger category of congenital diarrheal and enteropathy disorders (CoDEs), which are genetic diarrheal disorders that can present in the neonatal period (70). Certain inflammasomopathies can also present with infantile enterocolitis (71), while IEIs such as immunodysregulation polyendocrinopathy X-linked syndrome (IPEX) and X-linked inhibitor of apoptosis (XIAP) deficiency can also have similar intestinal symptoms in the neonatal period (72, 73). Persistent colitis or diarrhea is unusual in premature infants and should prompt genetic investigation for underlying etiology.

### Bronchopulmonary Dysplasia

BPD is a chronic lung disease that affects extremely premature infants and can be debilitating or fatal. Presentation and severity are widely variable (74). BPD can involve the airways, parenchyma, and vasculature. Precise mechanisms of disease are unknown, although long-term invasive mechanical ventilation and abnormal oxygen levels are known to be factors. Targeted panels are widely available to investigate known mutations in non-immune genes that cause severe neonatal lung disease (75). Patients with BPD frequently develop recurrent tracheitis and pneumonias with a variety of gram positive and negative organisms (76). Recurrent upper and lower airway infections represent well-known complications of IEI and are associated with secondary chronic pulmonary disease, although this typically develops years into the disease course (77). Immune dysregulation in children with IEI is also believed to mediate structural damage to the lungs over time, which can be life-limiting. Presentations are varied, with proximal and distal airways, lung parenchyma, and lung vasculature all affected (78).

While there is no known association between BPD and IEI, recurrent pulmonary infections in a patient with BPD are also a potential sign of IEI and may warrant further evaluation if unusually severe or if they feature an unusual pathogen (**Table 1**). In addition, mouse studies have shown that the hypoxic insult that occurs during BPD can result in thymic atrophy with loss of thymocytes (79–81). While this has not been thoroughly examined in human infants, a subset of former premature infants did exhibit sustained T cell lymphopenia in a retrospective case review, suggesting there may be human correlation (10). This does not imply that BPD causes IEI, but to say that IEI may be more complicated to screen for using standard lymphocyte test values in infants with BPD.

## Approaches/Solutions

Among those who contract infections in the NICU, premature infants cannot be distinguished from infants with IEI on the basis of clinical presentation or microbiology. The preterm neonate may exhibit a developmentally appropriate, but still abnormal, response to serious bacterial or fungal infection. The data discussed above support the notion that, in certain circumstances, clinical findings that are common in the premature neonate should prompt the clinician to consider screening for IEI. Given the substantial overlap in clinical symptomatology between premature infants and those with IEI, we advocate a conservative approach. Many other reviews have covered appropriate testing pathways for a range of IEIs (82–84). We will summarize briefly here but encourage clinicians to follow standard diagnostic approaches for symptoms that might suggest an IEI. For symptoms that overlap with autoinflammatory disorders, testing should include the standard acute phase reactant panels and cytokines including IL-18 levels and soluble IL-2R levels (53). Autoinflammatory gene panels can be sent if the first testing step is abnormal or inconclusive, and in fact given the clinical overlap of these disorders is important to send early (53, 85). For innate immune disorders the testing ordered should reflect the clinical scenario, for instance for a patient with a an intraperitoneal fungal ball would raise concern for chronic granulomatous disease and a dihydrorhodamine (DHR) test should be done and followed with CGD genetic testing if abnormal.

When there is concern for SCID or a combined immunodeficiency, which tends to be the most common scenario in premature infants, the first diagnostic steps should still be newborn screening with T cell receptor excision circles (TRECs) followed by flow cytometry and mitogen testing (**Table 1**). Furthermore, given the difficulty in interpreting flow in this population, where T cell lymphopenia is prominent, clinicians should adopt a more aggressive approach when evaluating for IEI if the results are equivocal. While a suggested reference range for flow cytometry in premature infants has been published (86), most commonly the challenge is interpreting whether abnormal results indicate an IEI or are explained by clinical circumstances, as these premature babies are often very ill and frequently on steroid therapy. If first line screening is abnormal or equivocal and substantial clinical evidence of an IEI is present (very severe infection, multiple infections, unusual organism, or unusual healing), we advocate that genetic testing be undertaken. A “wait and see” approach in this population could have devastating, sometimes fatal, consequences. Historically, there has been hesitancy to send extended testing in premature infants due to their small relative blood volume and the amount of blood needed for testing, but presently most children’s hospitals can do flow cytometry reliably on 1cc of blood or less. Genetic testing can often be done on oral saliva swabs, making blood draws even less of a concern.

Newborn screening for SCID has been of great benefit in the neonatal population. In premature infants, however, the false positive rate is high. In some states, such as Massachusetts, a conservative T cell receptor excision circle (TREC) threshold leads to an even higher rate of false positives in premature infants. As many more infants are referred for consultation and flow cytometry (87, 88), this leads to “TREC fatigue”, where low TRECs in premature infants are typically assumed to be due to immaturity or to critical illness, and the recommendation is to repeat testing every two weeks until either TRECs are normal, or the patients is more mature. In the original report of the first two years of California’s newborn screening for SCID, of the premature infants who tested positive initially, only 11% remained with abnormal TRECs by 3-4 weeks of life (89). If a state algorithm is to repeat NBS in premature infants, this should be time limited and not continue indefinitely. In the end, it is a disservice to premature infants to be continuously administering a screening test that will not be promptly acted upon. However, no clear alternatives to the TREC assay exist for premature infants. In fact, the scope of this problem is recognized in the community and the Association of Public Health Laboratories has been enrolling newborn screening programs in a quality improvement initiative to improve TREC standard ranges for premature infants (https://www.aphl.org/rfp/Pages/NBS-SCID.aspx). While it would neither be cost effective nor clinically effective to perform genetic testing on every premature infant with low TRECs, we recommend that patients who have had a history of abnormal TRECs and any serious or unusual infection have flow cytometry testing. If flow cytometry is inconclusive, even in the setting of illness or steroid use, the baby should be considered for whole exome sequencing (WES). A large number of tertiary children’s hospitals offer rapid WES for scenarios where testing will impact clinical management, with results available in as little as 7 days (90–92). Immunologists have already begun to advocate for more liberal use of genetic testing, even to the point of including genetic testing for IEI in newborn screening panels (93).

In sum, IEI and disorders of prematurity can present with similar features and are not mutually exclusive. Existing screening tests, especially for T cell-based disorders, have low specificity in the premature population. When a patient has a concerning clinical history, definitive genetic testing should be undertaken if immune testing is abnormal, unclear or equivocal, rather than waiting for the patient to be more mature. Clinicians need to have a high index of suspicion for IEI in premature infants, as we do not know the true incidence of IEI in this population. Finally, new diagnostic strategies or better data about normal test values in this population could be especially helpful for diagnosing IEI in the premature population.

## Author Contributions

Both authors contributed equally to the manuscript, including development of the proposal, research and writing. Both AO and SG edited the final version and approve of submission of the final version.

## Funding

This work was supported by the National Institutes of Health K08AI151265 (SG) and K08DK120871 (AO); Children’s Hospital of Philadelphia Research Institute (SG); the Manton Center for Orphan Disease Research (AO) and Children’s Hospital Boston Division of Newborn Medicine (AO).

## Conflict of Interest

The authors declare that the research was conducted in the absence of any commercial or financial relationships that could be construed as a potential conflict of interest.

## Publisher’s Note

All claims expressed in this article are solely those of the authors and do not necessarily represent those of their affiliated organizations, or those of the publisher, the editors and the reviewers. Any product that may be evaluated in this article, or claim that may be made by its manufacturer, is not guaranteed or endorsed by the publisher.
